# T-cell phenotypes in chronic rhinosinusitis with nasal polyps in Japanese patients

**DOI:** 10.1186/s13223-015-0100-2

**Published:** 2015-11-19

**Authors:** Shintaro Baba, Ryoji Kagoya, Kenji Kondo, Maho Suzukawa, Ken Ohta, Tatsuya Yamasoba

**Affiliations:** Department of Otolaryngology, Faculty of Medicine, Graduate School of Medicine, The University of Tokyo, 7-3-1 Hongo, Bunkyo-ku, Tokyo, 113-8655 Japan; Department of Otolaryngology, Tokyo Metropolitan Children’s Medical Center, Tokyo, Japan; Division of Respiratory Medicine and Allergology, Department of Medicine, Teikyo University School of Medicine, Itabashi-ku, Tokyo, Japan; National Hospital Organization Tokyo National Hospital, Kiyose, Tokyo Japan

**Keywords:** Treg, Th17, Th1/Th2, T cells, Nasal polyps

## Abstract

**Background:**

Chronic rhinosinusitis with nasal polyps is characterized by local inflammation and is categorized into two subtypes in Japan: eosinophilic chronic rhinosinusitis, and non-eosinophilic chronic rhinosinusitis. The objective of this study was to investigate the expression of key transcription factors for Treg and Th1/Th2/Th17 cells, in relation to the mRNA expression of representative cytokines in these two subtypes of chronic rhinosinusitis with nasal polyps.

**Methods:**

The expression of forkhead box P3 (FOXP3), T-box transcription factor (T-bet), GATA3, retinoid acid-related orphan receptor C (RORc), the suppressive cytokines TGF-β1 and IL-10, and Th1/Th2/Th17 cytokines (IFN-γ, IL-4, IL-5, IL-13, IL-17) were analyzed by means of RT-PCR in eosinophilic polyps. Eosinophilic polyps were defined as having an eosinophil count of more than 50 per microscopic field (×400 magnification) using five fields located in the subepithelial area of the polyps, while the non-eosinophilic polyps and controls did not fulfill this criteria. The numbers of T cells, CD4+ T cells, CD8+ T cells and Treg were histologically counted using sections that were immunostained for CD3, CD4, CD8, and FOXP3, respectively.

**Results:**

In eosinophilic polyps, we observed significantly fewer CD4+ T cells and CD8+ T cells, and lower GATA3, RORc and IL-10 mRNA expression, but a significantly higher IL-5, and IL-13 mRNA expression compared with controls, whereas FOXP3 and T-bet mRNA expression were not significantly different compared with controls. In non-eosinophilic polyps, FOXP3, IL-10, IL-17A, TGFβ1 and IFNγ mRNA expression was significantly higher compared with controls, whereas IL-4, 5 and 13 expression was not significantly different from controls.

**Conclusion:**

We showed a reduction of GATA3 and RORc mRNA, low Treg-related cytokines and elevated Th2 cytokine levels in eosinophilic chronic rhinosinusitis, whereas we demonstrated the upregulation of Treg cells and increases of Th1 and Th17 cytokines in non-eosinophilic chronic rhinosinusitis in the Japanese population. The different mRNA expression profiles of Treg and Th1/Th2/Th17 signature transcription factors and cytokines between eosinophilic chronic rhinosinusitis and non-eosinophilic chronic rhinosinusitis suggests heterogeneity in the pathogenesis of chronic rhinosinusitis with nasal polyps.

## Background

Chronic rhinosinusitis with nasal polyps (CRSwNP) is an inflammatory disease that remains difficult to treat despite advances in medical and surgical therapy. The majority of patients with CRSwNP in the United States and Europe have pronounced infiltration of eosinophils and expression of interleukin-5 (IL-5) in nasal polyps [1]. In contrast, greater heterogeneity in CRSwNP has been reported in East Asian countries, such as Japan and China. For example, Cao et al. reported that more than half of Chinese CRSwNP patients presented with a Th17-dominant, neutrophilic inflammation pattern [2]. In a recent study by Shi et al., they found significantly enhanced mucous metaplasia and tissue remodeling in non-ECRS patients in China [3]. More than half of CRSwNP cases in Japan do not exhibit eosinophil-dominant inflammation [4, 5]. In Japan, CRSwNP is categorized into two subtypes: eosinophilic chronic rhinosinusitis (ECRS), which is similar to the CRSwNP in western countries, and non-eosinophilic chronic rhinosinusitis (non-ECRS), which is characterized by Th1-dominant inflammation [4].

Naive T cells differentiate toward different T-cell subtypes on the basis of the expression of certain transcription factors. T-box transcription factor (T-bet) directs lineage commitment toward Th1 cells [6]; GATA-3 is critical for commitment toward Th2 cells and controls the expression of IL-4 and IL-5 [7, 8]. The balance between Th1 and Th2 is controlled by T regulatory (Treg) cells [9]. Forkhead box P3 (FOXP3) is essential for Treg development, survival, and function [10]. Recent studies indicate that Treg cells play an important role in the expression and regulation of T-cell subtypes in CRSwNP both in Asian [11] and Caucasian [12] regions.

Th17 cells are newly identified immune/inflammatory cell subsets which are now widely believed to be critical for the regulation of various chronic immune diseases [13]. Th17 cells are characterized by their preferential production of interleukins IL-17A and F and require retinoid orphan nuclear receptors (RORc) as a key transcription factor for their differentiation in humans [14, 15]. IL-17A has been revealed to play a significant role in regulating inflammation, modulating airway structural cells and stimulating innate immunity to mediate neutrophil recruitment in asthma and other airway diseases [16]. Recently, Xia et al. reported that IL-17A plays a crucial role in stimulating the production of MUC5AC and goblet cell hyperplasia through the act1-mediated signaling pathway, and that IL-17A may be a promising therapeutic target for the management of Th17-dominant CRSwNP patients in China [17].

Further understanding of the immunopathologic features of CRS in Asian populations will be of great value to elucidate the pathogenesis of CRS. In this study, we aimed to investigate the expression of key transcription factors for Treg and Th1/Th2/Th17 cells, in relation to the mRNA expression of representative cytokines, in ECRS and non-ECRS subgroups.

## Patients and methods

### Patients

The diagnosis of CRSwNP was based on the criteria of the EAACI position paper [1], which defined it as having two or more of the following symptoms: blockage/congestion, discharge, anterior/posterior drip, facial pain/pressure, reduction or loss of smell for at least 3 months and endoscopic signs of nasal polyp(s). Patients with CRSwNP associated with chronic obstructive pulmonary disease, diffuse panbronchiolitis, Churg-Strauss Syndrome, congenital mucociliary diseases or cystic fibrosis were excluded from this study. None of the patients included had been treated with systemic corticosteroids or other immune-modulating drugs for at least 1 month prior to surgery, although some patients had received antihistaminic agents and/or macrolide antibiotics.

Patients were classified into two groups: the ECRS group, which was defined as having an eosinophil count of more than 50 per microscopic field (×400 magnification) using five fields located in the subepithelial area of the polyps [4], and the non-ECRS group, which did not fulfill this criteria. The normal-appearing mucosa of the uncinate processes, which were surgically removed in eight patients without CRS (three with frontal sinus cysts, five with maxillary sinus tumors) served as the non-CRS control group. The study was approved by the ethical committee of The University of Tokyo Hospital (#2656). Informed consent was obtained from each patient before collecting samples.

### Sampling of tissue specimens and histological procedures

The nasal polyps and control mucosae were harvested during endoscopic sinus surgery. A part of each sample was fixed in 10 % formalin, embedded in paraffin, sectioned at 4 μm-thick, mounted on MAS-coated slides (Matsunami Glass, Osaka Japan) and used for hematoxylin-eosin staining as well as for immunohistochemistry. The remaining part was immediately immersed in RNA later^®^ (Life Technologies, Carlsbad, CA, USA) and used for real time PCR analysis. Unfortunately we were unable to harvest enough mucosal tissue in some cases and were therefore unable to carry out some RT-PCR analyses in these cases (see Results and Table 1).

**Table 1:** The number of inflammatory cells, expression of cytokines and transcription factors in normalized relative quantities (NRQ), described as median (IQR) in the tissue of nasal polyps of ECRS and non-ECRS patients, and normal controls

Nasal tissue analysis	Eosinophilic CRS polyps	Non-eosinophilic CRS polyps	Non-CRS controls	Multiple comparison	ECRS vs NC	ECRS vs non-ECRS	Non-ECRS vs NC
	Median (IQR)	Median (IQR)	Median (IQR)	P-value	P-value	P-value	P-value
The number of inflammatory cells							
Eosinophils	127.4 (81.4–180.7)	2.5 (0.55–7.3)	0 (0–0.05)	*<0.001*	*<0.001*	*<0.001*	*0.0082*
T cells (CD3)	29.8 (27.7–72.1)	60.5 (34.6–113.0)	56.2 (37.1–66.3)	0.34	0.52	0.14	0.74
CD4+ cells	3.4 (2.3–7.3)	16.4 (10.0–24.2)	13.3 (10.3–16.5)	*0.0016*	*0.005*	*0.0014*	0.42
CD8+ cells	36.6 (16.2–56.1)	63.2 (42.9–82.1)	34.4 (23.4–58.0)	*0.029*	0.67	*0.022*	*0.038*
mRNA expression of transcription factors							
Tbet	0.85 (0.5–1.4)	2.5 (1.2–113.2)	1.7 (1.1–2.7)	0.06	0.17	*0.03*	0.28
GATA3	0.56 (0.33–0.73)	1.2 (0.54–39.9)	1.0 (0.9–1.9)	*0.018*	*0.003*	*0.0049*	0.92
FOXP3	2.3 (1.9–3.2)	10.0 (6.2–30.8)	2.1 (1.0–4.5)	*<0.001*	1	*<0.001*	*0.005*
RORc	0.37 (0.096–0.49)	0.66 (0.48–10.9)	1.1 (0.91–1.56)	*0.007*	*0.0025*	*0.0023*	0.56
mRNAexpression of key cytokines							
IFN-γ	0.77 (0.28–1.2)	5.2 (1.4–16.4)	1.4 (0.81–2.2)	*0.003*	0.25	*0.0015*	*0.04*
IL-5	137.8 (21.3–573.5)	0.11 (0.0–1.1)	0.39 (0.069–1.0)	*<0.001*	*<0.001*	*<0.001*	0.88
IL-13	16.5 (3.9–61.3)	0.3 (0.0–4.5)	0.81 (0.11–3.6)	*<0.001*	*0.006*	*<0.001*	0.67
IL-4	0.62 (0.19–0.98)	0.3 (0.095–0.89)	0.78 (0.17–1.3)	0.58	0.79	0.39	0.38
IL-4R	0.052 (0.036–0.88)	0.11 (0.067–0.27)	0.24 (0.11–0.67)	*<0.001*	*<0.001*	*0.03*	*0.04*
IL-10	5.3 (2.5–8.1)	8.9 (4.0–268.4)	1.0 (0.93–2.4)	*0.005*	*0.04*	0.13	*0.002*
TGF-β1	2.9 (1.9–4.0)	6.1 (3.6–17.6)	2.4 (1.5–2.9)	*0.007*	0.28	*0.02*	*0.005*
IL-17	4.1 (1.3–12.3)	22.0 (8.3–4534.0)	4.0 (1.6–8.2)	*0.042*	0.92	*0.03*	*0.03*

P < 0.05 were considered to indicate statistical significance (italic values)

### Immunohistochemistry

The following primary antibodies were used for identifying inflammatory cells in the specimens: anti-CD3 (rabbit monoclonal, clone SP7; Nichirei, Tokyo, Japan), anti-CD4 (rabbit monoclonal, clone SP35; Acris Antibodies Inc, San Diego, California, USA), anti-CD8 (mouse monoclonal, clone C8/144B; Nichirei), and anti-FOXP3 (mouse monoclonal, clone 236A/E7; Abcam, Tokyo, Japan).

Sections rehydrated through a xylene and ethanol series were immersed in 10 mM citrate buffer solution (pH6.0, Dako Cytomation Japan, Kyoto, Japan) and autoclaved at 121 °C for 20 min for the retrieval of antigens. Endogenous peroxidase activity was blocked by treatment with 10 % hydrogen peroxide in methanol for 15 min at RT. Next, sections were incubated for 30 min in a blocking solution (PBS, pH 7.4, containing 2 % bovine serum albumin (Sigma-Aldrich Japan), 0.1 % Triton X-100, and 0.1 % sodium azide) at RT to reduce nonspecific antibody binding, then incubated with: anti-CD3 antibody (1:400 in blocking solution), anti-CD4 antibody (1:100 in blocking solution), anti-CD8 antibody (1:100 in blocking solution) and anti-FOXP3 antibody (1:100 in blocking solution) overnight at 4 °C. These antigen retrieval and incubation conditions were determined based on the results of preliminary experiments (data not shown). After several washes in PBS (pH 7.4), sections were incubated for 30 min at RT with horseradish peroxidase (HRP) conjugated with anti-mouse or rabbit IgG antibodies (Simplestain MAX-PO, (M) and (R), ready-to-use; Nichirei, Tokyo, Japan) corresponding to the primary antibodies. After more washes with PBS (pH 7.4), immunoreactivity was made visible with diaminobenzidine (DAB) (Simplestain DAB, ready-to-use; Nichirei). After washing with distilled water, the sections were counterstained with hematoxylin, then dehydrated and mounted. To ensure that there was no non-specific staining of primary antibodies, the primary antibodies were omitted from the reaction.

### Cell counting

To determine the degree of eosinophil infiltration in the tissues, two of the authors independently manually counted the number of infiltrated cells in five random fields using H-E stained sections under light microscopy at high magnification (×400) in a blinded manner. The numbers of T cells, CD4+ T cells, CD8+ T cells and Treg were counted in the same manner using sections that were immunostained for each cell type.

### Real-time quantitative PCR analysis

The sample tissues were lysed in ISOGEN (Nippon Gene, Tokyo, Japan) and the total RNA was extracted according to the manufacturer’s instructions. The mRNA expression was analyzed using an Applied Biosystems 7500 Real Time PCR System (PE Applied Biosystems, Foster City, CA, USA). The primers and probes for human β-actin, IL-4, 5, 13 and IL-4 receptor were designed by PE Applied Biosystems and their mRNA expression analysed using the TaqMan detection system. The mRNA of the transcription factors FOXP3, GATA3, T-bet, and RORc and cytokines IL-10, IL-17A, TGF-β1, and IFN-γ were analyzed using the SYBR Green detection system. The primers and probes for these transcription factors and cytokines were designed by SABiosciences (Hilden, Germany). Nuclease-free water was substituted for cDNA in negative controls.

For relative quantification, data were analyzed by the ΔΔC_t_ method and normalized to the average of housekeeping genes such as β-actin (ACTB) in the TaqMan detection system and glyceraldehyde-3-phosphate dehydrogenase (GAPDH) [18] in the SYBR Green detection system. For each sample using the TaqMan detection system, the differences in threshold cycles between the cytokine and β-actin or GAPDH genes (ΔC_t_ sample, ΔC_t_ control) were determined, and a calibrated ΔC_t_ value (ΔΔC_t_, ΔC_t_ sample − ΔC_t_ control) was calculated. Samples using the SYBR Green detection system were normalized to the average of the housekeeping gene GAPDH. Then the relative quantitation (RQ) values were calculated using the following equation: RQ = $2^{{ - \Delta \varDelta {\text{C}}_{\text{t}} }}$.

### Statistical analyses

Statistical analyses were carried out using SPSS statistical software (SPSS, Chicago, IL, USA). All data are expressed as mean ± standard error in each group. When comparisons were made between groups, the Kruskal–Wallis test was used to assess significant intergroup variability. The significance of the difference between groups was determined using the Mann–Whitney U test. A difference was considered significant if p < 0.05.

## Results

### Patient profiles

The ECRS group included 15 male patients (age range 42–76 years, mean age 58.3 years), in which the average eosinophil count in the total white cell count in peripheral blood was 6.8 % (range 3.1–12.8 %) and the average number of eosinophils was 399.0/mm^3^ (range 210.8–625.6/mm^3^). Seven patients in this group had allergic rhinitis, one had asthma, while eight reported no allergy-related diseases. All twelve of the female ECRS patients identified on the hospital database has used topical or oral steroids within a month prior to surgery and therefore had to be excluded from the present study.

The non-ECRS group included 16 patients (5 females and 11 males, age range 40–70 years, mean age 57.6 years), in which the average eosinophil/total white cell count in peripheral blood was 1.9 % (range 0.4–5.2 %) and the average number of eosinophils was 121.4/mm^3^ (range 40.0–260.0/mm^3^). Thirteen of these patients did not have any allergy-related diseases, while the other three had allergic rhinitis. No patients in the non-ECRS group had asthma or aspirin sensitivity. The non-CRS group included 8 patients (3 females and 5 males, age range 30–69 years, mean age 52.6 years), in which the average eosinophil count in the total white cell count in peripheral blood was 2.2 % (range 1.0–3.7 %) and the average eosinophil number was 126.5/mm^3^ (range 50.0–210.9/mm^3^). Six of these patients did not have any allergy-related diseases while two had allergic rhinitis. No patients in the non-CRS group had asthma or aspirin sensitivity. There was no significant difference in age among three groups, whereas the peripheral blood eosinophil count was significantly greater (p < 0.001) in the ECRS group compared with the non-ECRS group and non-CRS control group. The patient profiles are shown in Table 2.

**Table 2:** Patient profiles

Patient group	Number (n)	Male:female	Age (years)^a^	Asthma	Allergic rhinitis	Eosinophil count in the total white cell count (%)^a^	Eosinophil count in nasal tissues^b^
ECRS	15	15:0	58.3 (42–76)	1/15	7/15	6.8 (3.1–12.8)	127.6 (53.2–385.2)
Non-ECRS	16	11:5	57.6 (40–70)	0/16	3/16	1.9 (0.4–5.2)	2.5 (0–47.8)
Non-CRS	8	5:3	52.6 (30–69)	0/8	2/8	2.2 (1.0–3.7)	0.0 (0–3.8)

^a^Data are expressed as means and ranges

^b^Data are expressed as medians and ranges

**Fig. 1:**
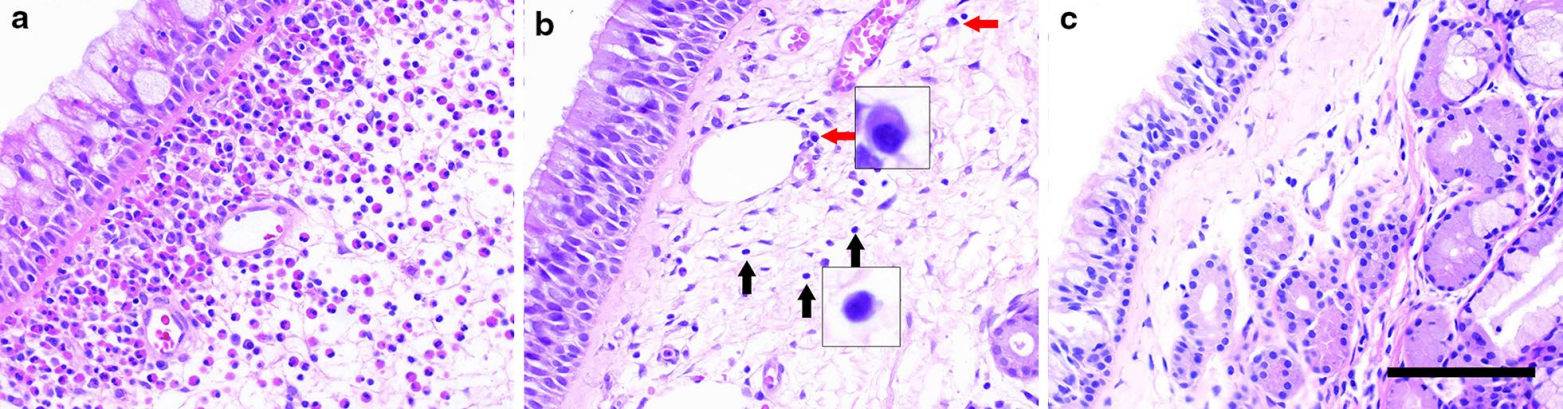
Representative H-E-stained histological sections of nasal polyps (**a**, **b**) and uncinate process mucosa (**c**) obtained from ECRS (**a**), non-ECRS (**b**) and non-CRS (**c**) groups. In the ECRS group, almost all of the infiltrating cells are eosinophils (**a**), whereas most of the infiltrating cells are lymphocytes (*black arrows*) and plasma cells (*red arrows*) in the non-ECRS group (**b**). Few inflammatory cells are infiltrating in non-CRS group (**c**). *Scale bar* 100 μm

Histological observations of the nasal polyps showed that eosinophils were the predominant type of infiltrating cell in the ECRS group (Fig. 1a). On the other hand, most of the infiltrating cells were lymphocytes and plasma cells in the non-ECRS group (Fig. 1b) while there were few infiltrating inflammatory cells in the non-CRS group (Fig. 1c). We identified the type of inflammatory cell based on their morphology in H-E stained sections. The pattern of inflammatory cell infiltration in the non-ECRS group was similar to that reported in our previous study in which an increased number of B cells and plasma cells was demonstrated by using immunohistochemistry for cell-type-specific markers [19].

### Immunohistochemical analysis of inflammatory cells in nasal tissues

**Fig. 2:**
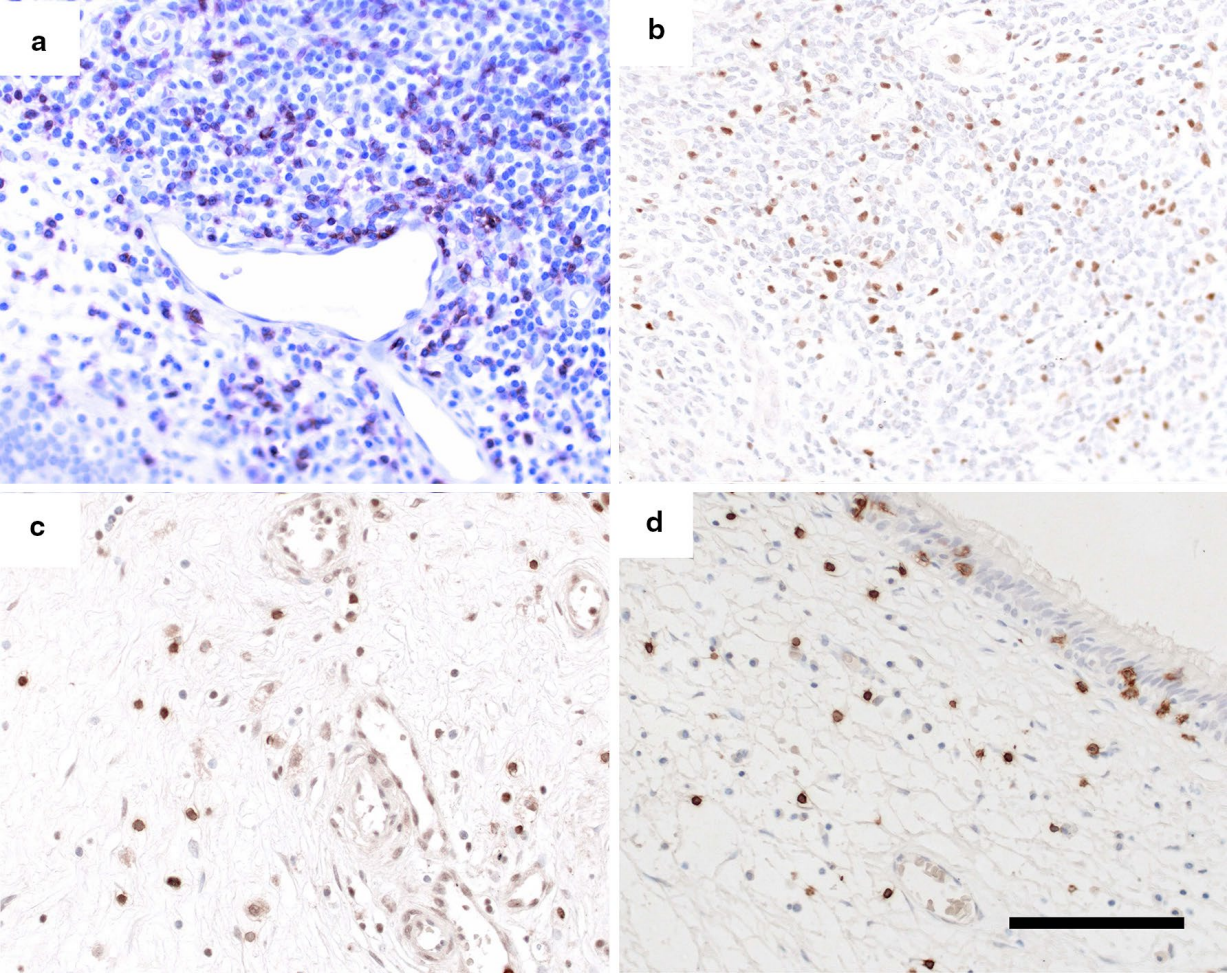
Photomicrographs showing immunohistochemical staining for CD3 (T-cells; **a**), FOXP3 (**b**), CD4 (**c**) and CD8 (**d**) in non-ECRS polyps. *Scale bar* 100 μm

**Fig. 3:**
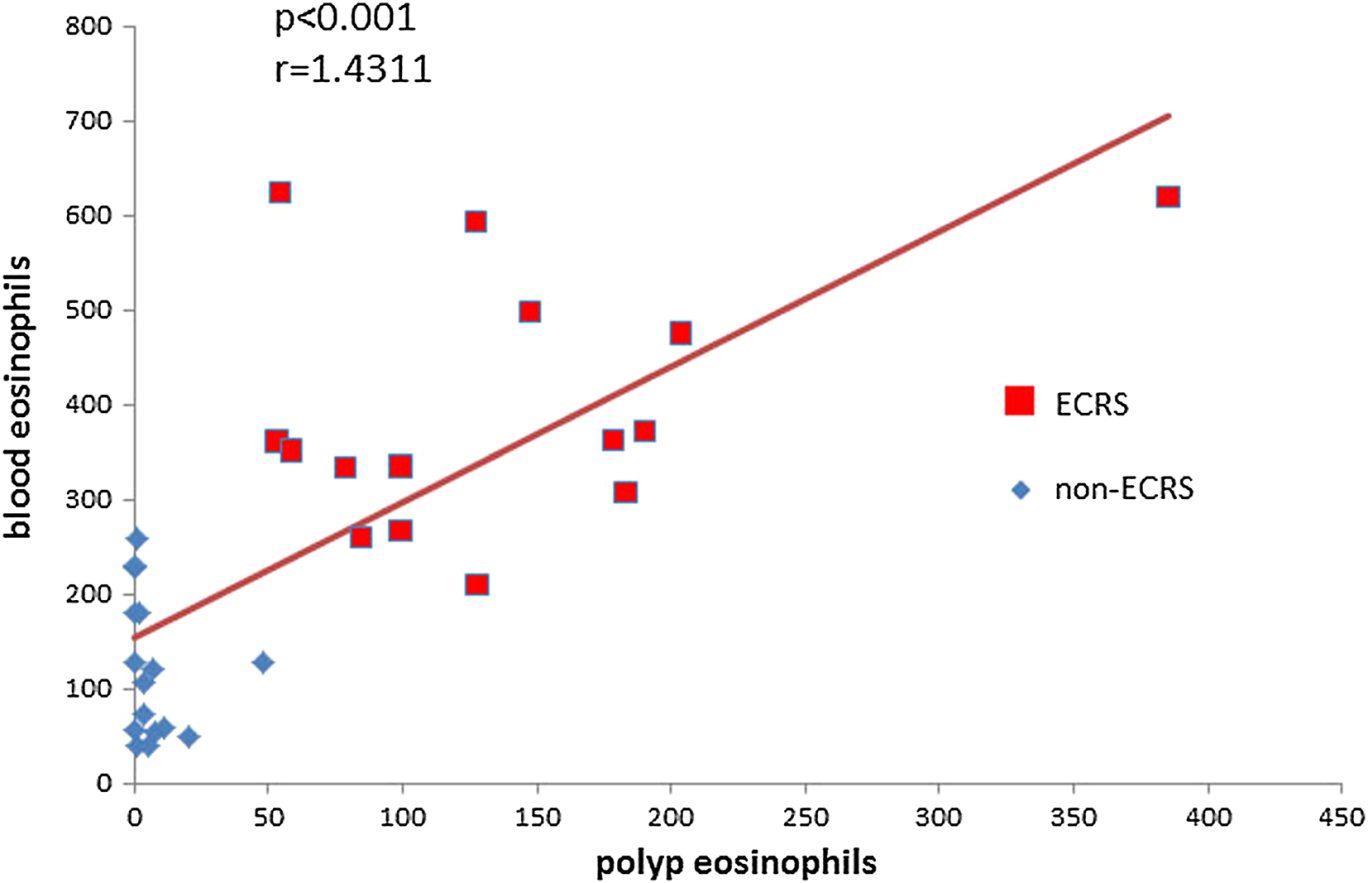
Correlation between the number of eosinophils in polyps and the number of blood eosinophils in the ECRS group (*red squares*) and non-ECRS group (*blue diamonds*)

The number of eosinophils and cells positive for CD3 (T-cells), CD4 and CD8 were counted using H-E and immunohistochemical staining in ECRS polyps, non-ECRS polyps and non-CRS controls. Typical photomicrographs of immunohistochemically stained nasal tissue are shown in Fig. 2. As illustrated in Table 1, the median and interquartile range counts for eosinophils were significantly higher in ECRS polyps (n = 15, median 127.6; range 53.2-385.2) compared with non-CRS controls (n = 8, median 0.0; range 0.0–3.8; p < 0.001) and non-ECRS polyps (n = 16, median 2.5; range 0.0–47.8; p < 0.001) (Table 1). The number of eosinophils in polyps had a significant correlation with the number of blood eosinophils in both ECRS and non ECRS polyps (n = 31, r = 1.4311, p < 0.0001; Fig. 3).

**Fig. 4:**
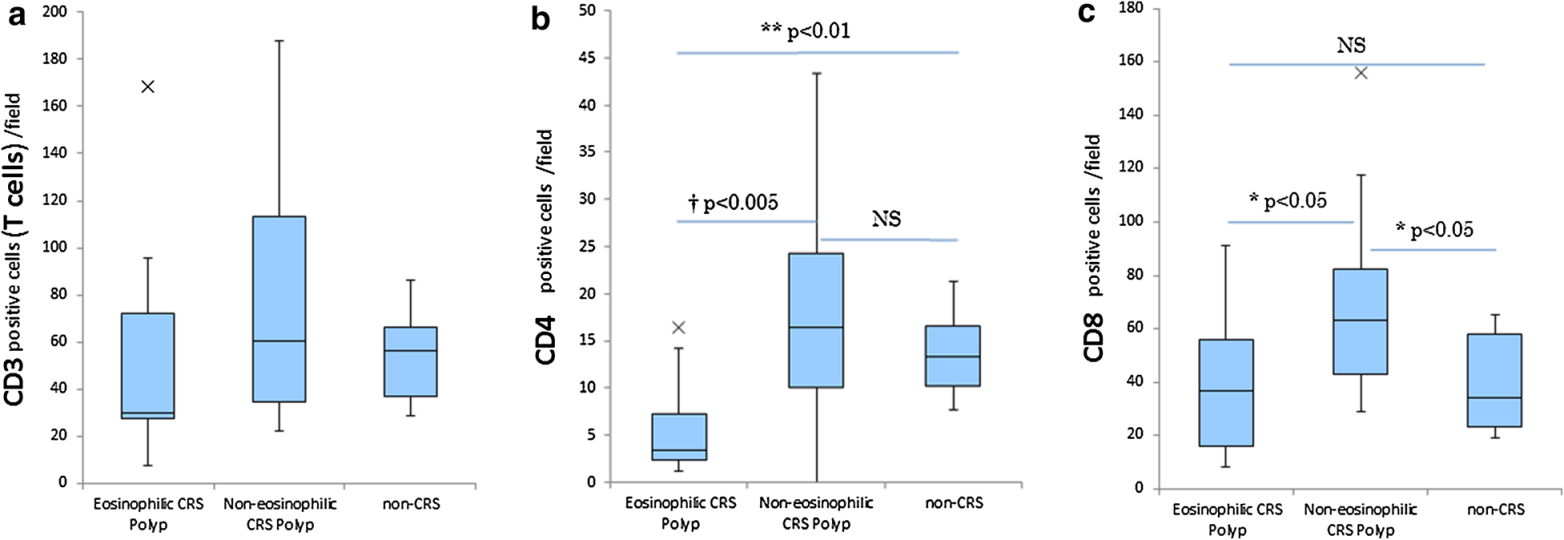
The number of inflammatory cells in mucosal specimens. The number of CD3 positive cells (T cells) (**a**), CD4 positive cells (**b**) and CD8 positive cells (**c**) per field in the ECRS and non-ECRS polyps, as well as the non-CRS controls. Data in *box-and-whisker plots* represent the median, lower and upper quartile and the minimum to maximum value. ×, outliers (^†^p < 0.005, **p < 0.01, *p < 0.05)

As we have previously reported [20], the median counts for CD4+ T cells and CD8+ T cells were significantly higher in non-ECRS polyps (n = 15, CD4: median 16.4; range 0–43.4, CD8: median 63.2; range 28.8–155.8) compared with ECRS polyps (n = 12, CD4: median 3.4; range 1.2–16.4; p < 0.005, CD8: median 36.6; range 8.4–91.4; p < 0.005) (Fig. 4b, c; Table 1). Moreover, there were significantly fewer CD4+ T cells in ECRS polyps than in non-CRS controls (n = 7, CD4: median 13.3; range 7.8–21.3; p < 0.01). No significant difference was observed in the median count for T-cells among the groups (p > 0.05) (Fig. 4a; Table 1).

### Immunohistochemical localization of FOXP3

Representative microphotographs of non-ECRS polyps immunostained for FOXP3 are shown in Fig. 2b. FOXP3 was localized in cells in the subepithelial layer, cells around the capillary vessels and cells in the lymphoid follicles of ECRS and non-ECRS polyps.

### Tissue expression of Treg and Th1/Th2/Th17- transcription factors

**Fig. 5:**
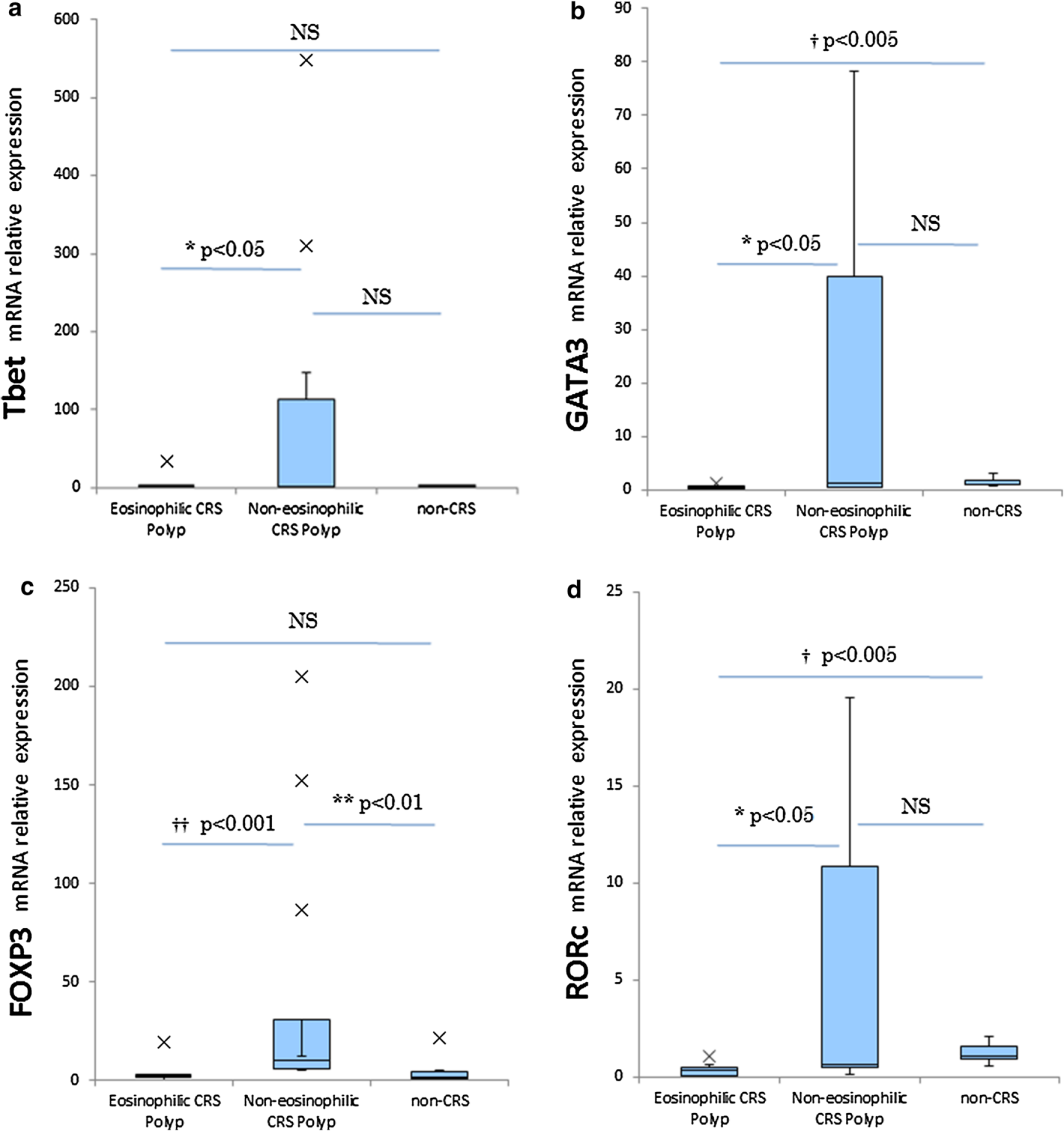
mRNA expression of transcription factors (T-bet, GATA-3 FOXP3, RORc) involved in naive T-cell differentiation in ECRS, non-ECRS and non-CRS controls. The *box* represents the distribution of values; the *line* across the *box* represents the median; the box stretches from the lower hinge (the 25th percentile) to the upper hinge (the 75th percentile). ×, outliers (^††^p < 0.001, ^†^p < 0.005, *p < 0.05)

The expression levels of key transcription factors for Treg (FOXP3), Th1 (T-bet), Th2 (GATA3), and Th17 (RORc) were directly analyzed on tissue biopsies by means of quantitative real-time PCR. This revealed a significantly lower GATA3 mRNA expression level in ECRS polyps (n = 10) compared with non-ECRS polyps (n = 10, p < 0.05) and controls (n = 7, p < 0.005) (Fig. 5b; Table 1). The expression of RORc was significantly lower in ECRS polyps (n = 10) compared with non-ECRS polyps (n = 10, p < 0.05) and controls (n = 7, p < 0.005) (Fig. 5d; Table 1). T-bet was not significantly different between non-ECRS polyps (n = 10) and controls (n = 7), but was significantly higher in non-ECRS compared with ECRS polyps (n = 10, p < 0.05) (Fig. 5a; Table 1). Moreover, in non-ECRS polyps (n = 10), FOXP3 mRNA expression was higher compared with controls (n = 7, p < 0.01), and significantly higher compared with ECRS polyps (n = 10, p < 0.001) (Fig. 5c; Table 1).

### Treg- and Th1/Th2/Th17-related cytokine expression

Differences in Th1/Th2/Th17 cytokine patterns and Treg related TGF-β1/IL-10 were assessed at the mRNA level in ECRS polyps, non-ECRS polyps, and controls (Table 1). In ECRS polyps (n = 15), we found significantly higher mRNA expression of the Th2 cytokine IL-5 compared with controls (n = 8, p < 0.001) and with non-ECRS polyps (n = 13, p < 0.001) and a significantly higher mRNA expression of IL-13 in ECRS polyps (n = 15) compared with controls (n = 8, p < 0.01) and with non-ECRS polyps (n = 16, p < 0.001). For IL-4, no significant differences were noted among controls, ECRS, and non-ECRS polyps. Conversely, mRNA levels of IL-4 receptor (the common receptor of IL-4 and IL-13) were significantly lower in ECRS polyps (n = 14) compared with controls (n = 8, p < 0.05) and with non-ECRS polyps (n = 14, p < 0.05). In contrast, mRNA levels of the Th1 cytokine IFN-γ were significantly higher in non-ECRS polyps (n = 12) compared with controls (n = 6, p < 0.05) and with ECRS polyps (n = 10, p < 0.005). mRNA expression of TGF-β1 was significantly higher in non-ECRS polyps (n = 10) compared with controls (n = 7, p < 0.005) and with ECRS polyps (n = 10, p < 0.05). mRNA expression of IL-10 was significantly higher in non-ECRS polyps (n = 12) compared with controls (n = 7, p < 0.005). Furthermore, IL-10 mRNA levels were significantly lower in ECRS polyps compared with controls (p < 0.05). The mRNA expression of IL-17A was significantly higher in non-ECRS polyps (n = 10) compared with controls (n = 7, p < 0.05) and with ECRS polyps (n = 10, p < 0.05).

## Discussion

In this study, we analyzed the expression of the transcription factors FOXP3, T-bet, GATA3, and RORc in relation to Treg and Th1/Th2/Th17-related cytokines in the sinonasal mucosal tissue of ECRS polyps, non-ECRS polyps and non-CRS controls. We demonstrated a significantly lower mRNA expression of RORc in ECRS polyps compared with non-ECRS polyps and non-CRS controls in the Japanese population, indicating the reduction of Th17 effector cells in ECRS. Unexpectedly, we also found a significantly lower mRNA expression of GATA3 in ECRS polyps. In addition, we observed an increase in mRNA expression of FOXP3 in non-ECRS polyps compared with ECRS polyps and controls, and an increase in mRNA expression of T-bet in non-ECRS polyps compared with ECRS polyps, suggesting upregulation of Th1 and Treg cells in non-ECRS polyps. To the best of our knowledge, this report is the first to examine T-cell signature transcription factors and cytokines in nasal polyps comparing ECRS and non-ECRS in an Asian population.

Increased IL-17A has been proposed as being responsible for enhanced tissue neutrophilia, collagen deposition and corticosteroid resistance in polyp tissues [21, 22]. Furthermore, in vitro studies have shown that the induction of metalloproteinases by IL-17, which can lead to the characteristic edema formation in the nasal polyps of patients with cystic fibrosis, is characterized by abundant neutrophils and increased concentrations of IL-8 and MPO [23]. In addition to Th17 cells, other cell types also express IL-17 [24, 25]. CD8+ T cells, γδ T cells, invariant natural killer T cells, and lymphoid tissue inducer-like cells have all been found to express and produce IL-17 [24]. Treg cells can produce IL-17 when they are strongly activated in the presence of proinflammatory cytokines, such as IL-1β and IL-6 [25]. Therefore, increased IL-17A expression does not directly support the effect of Th17 cells. However, IL-17A and IL-17F are expressed most abundantly by Th17 cells [25]. In this study we demonstrated increased IL-17A mRNA expression in non-ECRS tissue. This finding, together with the upregulation of RORc, suggests a role of Th17 cells in the pathogenesis of non-ECRS.

Due to the absence or low expression of IL-17A in polyp tissues in western patients [26], Th17 cells have not been well documented in the pathogenesis of CRSwNP. Recently, some studies have demonstrated that Th17 cells are specifically increased in nasal polyp patients in China and eastern Asian regions compared to their western counterparts [17, 27]. Our results regarding Treg/Th1 ⁄ Th2 ⁄Th17 signature transcription factor profiles and IL-17 are partially discrepant from these findings. Zhang et al. [27] demonstrated that although IL-17 levels were obviously upregulated in nasal polyps from Chinese patients, RORc expression was not significantly different between patients and controls. Shen et al. [28] reported an increase in Th17 and a decrease in Treg in non-ECRS patients in China. Conversely, Li et al. [29] found that Th17 (RORc) and Th2 (GATA3) were downregulated and that Treg (FOXP3) was increased in non-ECRS patients in China. Meanwhile, Van Bruaene et al. [12] reported that for IL-17 and RORc expression in polyp tissue, no significant differences were noted between European patients and controls. We presume that these contradictory observations might be attributed to different ethnic groups and variations in the clinical severity of NP patients.

TGF-β1 induces FOXP3 expression in CD25- naive T cells to enforce transition to Treg cells and is a critical factor in the development of peripheral Treg cells [30]. We found a significant upregulation of TGF-β1 at the mRNA level in non-ECRS polyps compared with ECRS polyps and controls. Furthermore, high TGF-β1 mRNA levels coincided with adequate FOXP3 expression in non-ECRS tissue, implying a functional link. The effect of local TGF-β1 on Treg function has to be confirmed in non-ECRS tissue. In contrast, there was no significant difference in TGF-β1 mRNA expression in ECRS tissue versus controls.

IL-10, a cytokine that is intimately involved in the regulation of inflammatory and immune responses, was measured at the mRNA level. The induction of peripheral regulatory T cells by IL-10 points toward a crucial role in the establishment of peripheral tolerance [31]. Specifically, IL-10 is instrumental in Treg-mediated suppression of proliferation and cytokine production of naive CD4+ CD25-T, Th1, and Th2 cells. Activation of T cells in the presence of IL-10 induces a long-lasting state of nonresponsiveness or anergy [32]. Here, we found a significant upregulation of IL-10 at the mRNA level in non-ECRS tissue compared with controls, whereas IL-10 mRNA was downregulated in ECRS patients compared with controls. TGF-β and IL-10 are both suppressor cytokines that frequently occur together at sites of inflammation, and both cytokines cooperate in the resolution of inflammation. TGF-β can induce IL-10 production, and IL-10 facilitates TGF-β regulatory activity [33]. The striking coincidence of low IL-10 RNA levels, together with basal levels of TGF-β1 mRNA (not raised compared with controls) in ECRS tissue, points to the lack of a regulatory effect in the resolution of inflammation, hence contributing to the chronicity of this disease.

T-bet is a Th1-specific T-box transcription factor that controls the expression of the hallmark Th1-cytokine IFN-γ, and T-bet expression correlates with IFN-γ expression in Th1 cells [6]. We report an increase in mRNA expression of IFN-γ in non-ECRS compared with ECRS tissues and controls and an increase in mRNA expression of T-bet in non-ECRS compared with ECRS tissues. Different from T-bet, the expression of which is mainly restricted to the Th1 cell type, IFN-γ can be produced by several cell types including CD4+ Th1 cells, CD8+ cells, and natural killer cells, but also B cells [6]. In this study, CD4+ and CD8+ T-cell levels were significantly decreased in ECRS patients compared with non-ECRS patients. Additionally, we have previously reported an increased number of plasma cells and increased expression of IgG mature transcripts in the same non-ECRS patients [19]. These data indicate that IFN-γ may contribute to the development of chronic inflammation in patients with non-ECRS.

The present study demonstrated significantly elevated IL-5 and IL-13 levels in ECRS polyps, although CD4+ cells and expression of Th2 signature transcription factor GATA3 mRNA were decreased. This was the most surprising result in our analysis and is difficult to interpret. The most straightforward explanation for lower GATA3 expression would be the decreased number of Th2 cells. However, the regulation of gene expression is a complex process, and both GATA3 and Th2 cytokines, originally regarded as indicators of Th2 cells, are actually expressed in other inflammatory cell types [34]. Therefore, the mRNA expression of GATA3 alone does not lead to any conclusion as to the number or activity of Th2 cells.

The expression of GATA3 mRNA in nasal polyps reported previously varies from paper to paper. In a Chinese population, Li et al. [29] reported that GATA3 mRNA expression was downregulated in polyp tissue, while Zhang et al. [27] reported that GATA3 expression in polyp tissue was not significantly different compared to controls. Conversely, Shi et al. [11] demonstrated that GATA3 expression was obviously upregulated in nasal polyps compared with the controls in a Chinese population. As for the western population, Van Bruaene et al. [12] reported that GATA3 expression was obviously upregulated in nasal polyps compared with controls and tissue from CRS patients without nasal polyps. Zhang et al. [27] also found GATA3 expression was upregulated in nasal polyps in a Belgian population. On the other hand, Miljkovic et al. [35] reported downregulated mRNA expression of GATA3 in nasal polyps from western patients compared with controls.

In our study, if we simply compared CRSwNP patients (which includes both ECRS and non-ECRS patients, n = 20) and controls, there was no significant difference in GATA3 expression between them (p = 0.11). The divergent data regarding GATA3 mRNA expression among these studies may therefore be more associated with the way patients were selected and categorized and less so with their ethnic background or variations in their clinical severity.

One explanation for the upregulation of Th2 cytokine in spite of decreased GATA3 expression may be an involvement of Group 2 innate lymphoid cells (ILC2s). Innate lymphoid cells (ILCs) are a heterogeneous group of cell types that play a role in innate immune responses and tissue remodeling at mucosal barrier surfaces. They are functionally related to T helper subsets, mirroring the production of specific T helper cytokines and having a lymphoid morphology. A subset of ILCs, termed group 2 ILCs (ILC2s), was recently identified in human nasal tissues [36, 37], and have been implicated in the initiation and coordination of Th2-type immune responses [38] and shown to produce the cytokines IL-5, IL-6, IL-9 and IL-13 [36, 38]. Very recently, Miljkovic et al. reported an upregulation of IL-5 and IL-13 mRNA levels and diminished mRNA expression of IL-25 and GATA3 in CRSwNP [35]. They also found that ILC2 s were significantly enriched in nasal polyp (CRSwNP) patients [35]. Moreover, Klein Wolterink et al. showed that the novel population of ILC2 surprisingly constitutes an important fraction of IL-5 and IL-13 cytokine producing cells, next to the classical Th2 cells, in allergen-induced allergic asthma in mice [39]. Therefore, ILC2 might be a major source of IL-5 and IL-13 instead of Th2 in ECRS polyps. Further research into the sources of Th2-inducing cytokines is warranted.

Our results demonstrate that regardless of the different T cell inflammatory patterns, both ECRS and non-ECRS patients showed the same overgrowth of nasal mucosa that characterizes CRSwNP patients. It may therefore be speculated that CRSwNP is not a simple, helper T cell-derived inflammatory disease, but involves other as yet unspecified pathological processes, such as impaired remodeling processes. With this possibility in mind, it may be necessary to develop an additional strategy to investigate the pathogenesis of nasal polyposis.

## Conclusion

Our findings show a reduction of GATA3 and RORc mRNA, low Treg-related cytokines and elevated Th2 cytokine levels in ECRS patients. In contrast, we demonstrated the upregulation of FOXP3 and RORc mRNA and increases of Treg, Th1 and Th17 cytokines in non-ECRS patients. This heterogeneity of immunological profiles in Japanese CRS patients suggests that the pathogenesis of nasal polyps in the Japanese population is not identical to that of nasal polyposis in Western patients, a fact which may be helpful in the development of novel therapeutic strategies.

## Abbreviations

CRS: Chronic rhinosinusitis
CRSwNP: Chronic rhinosinusitis with nasal polyps
ECRS: eosinophilic chronic rhinosinusitis
IL: interleukin
T-bet: T-box transcription factor
FOXP3: forkhead box P3
RORc: retinoid acid-related orphan receptor C
GAPDH: glyceraldehyde-3-phosphate dehygrogenase
TGF-β1: transforming growth factor-β1
ILCs: Innate lymphoid cells
IFN: interferon
